# Intra- and Inter-Regional Priming of Ipsilateral Human Primary Motor Cortex With Continuous Theta Burst Stimulation Does Not Induce Consistent Neuroplastic Effects

**DOI:** 10.3389/fnhum.2018.00123

**Published:** 2018-03-29

**Authors:** Michael Do, Melissa Kirkovski, Charlotte B. Davies, Soukayna Bekkali, Linda K. Byrne, Peter G. Enticott

**Affiliations:** Cognitive Neuroscience Unit, School of Psychology, Deakin University, Geelong, VIC, Australia

**Keywords:** corticospinal excitability, metaplasticity, motor cortex, neuroplasticity, theta burst stimulation, transcranial magnetic stimulation

## Abstract

Human responses to non-invasive brain stimulation (NIBS) techniques can be highly variable. Recently, priming protocols involving a conditioning round of NIBS applied to a target brain region prior to the application of a test protocol have shown promise in inducing more reliable effects. We investigated whether intra- or inter-regional priming of the left primary motor cortex (M1) using continuous theta burst stimulation (cTBS) can induce consistent, and reliable modulation of corticospinal excitability. Twenty healthy adults (six males) underwent four cTBS protocols. For intra-regional priming, cTBS was applied twice to the left M1 (M1-M1). For inter-regional M1 priming, cTBS was applied to the ipsilateral (left) dorsal premotor cortex (dPMC-M1), and ipsilateral (left) dorsolateral prefrontal cortex (DLPFC-M1). In the control condition, sham stimulation was applied to left M1, followed by active cTBS also applied to the left M1 (sham-M1). Each round of cTBS was separated by 10 min. Neuroplastic responses were indexed using motor evoked potentials (MEPs) elicited from the left M1 hand region, and measured from the contralateral first dorsal interosseous (right hand). MEP measurements were taken before the first round of cTBS priming, then immediately, 10, 20 and 30 min after the second test round of cTBS. The primary two-way repeated measures ANOVA revealed no significant differences in MEP responses across each condition (no main effects or interaction). Intra- and inter-regional priming of the left M1 using cTBS does not induce consistent neuroplastic effects. Further work is required to identify factors which contribute to such variability in human responses to NIBS.

## Introduction

Neuroplasticity refers to the brain’s capacity for neural restructuring, and underlies our capacity to acquire knowledge, retain memories, and to recover from injury and trauma (Hebb, 1949; Pascual-Leone et al., 2005). Some of the most well characterized forms of neuroplasticity include long-term depression (LTD) and long term potentiation (LTP; Malenka and Bear, 2004). In the human neocortex, similar LTD- and LTP-like effects can be induced using non-invasive brain stimulation (NIBS) techniques (Fitzgerald et al., 2006; Ridding and Ziemann, 2010; Chung et al., 2016). Correspondingly, a large body of work is now dedicated to the application of NIBS for investigative, and clinical applications (Wassermann and Lisanby, 2001; Daskalakis, 2005; Guse et al., 2010; Jaafari et al., 2012).

Although NIBS techniques can modulate corticospinal excitability, the neuroplastic effects reported in humans are often highly variable, thus limiting their potential utility (Ridding and Ziemann, 2010; Pell et al., 2011; Hamada et al., 2012; Goldsworthy et al., 2014). One method that may induce more homogenous and predictable effects involves “priming” a target region with a conditioning bout of NIBS before administration of the test protocol (Karabanov et al., 2015). Recently, Goldsworthy et al. (2012, 2015) have shown that two rounds of continuous theta burst stimulation (cTBS; a NIBS technique involving the delivery of very high frequency electromagnetic pulse bursts in the theta range) applied intra-regionally to the human primary motor cortex (M1) can induce strong, and persistent suppression of corticospinal excitability lasting up to 2 h.

Another approach which may yield effective protocols for inducing robust neuroplastic effects is through inter-regional priming (Karabanov et al., 2015; Müller-Dahlhaus and Ziemann, 2015). In general, the effects of intra- and inter-regional priming using NIBS techniques can be described using the Bienenstock, Cooper and Munro (BCM) theory of homeostatic metaplasticity (Bienenstock et al., 1982; Karabanov et al., 2015). One of the predictions made by the BCM model is the inhibitory effects of an LTD-inducing paradigm can be reversed into facilitation if it is preceded by low-frequency stimulation (i.e., a LTD paradigm). For example, Pötter-Nerger et al. (2009) found that the usual inhibitory LTD-like effects on M1 corticospinal excitability induced by paired associative stimulation (PAS_N20–5 ms_; a NIBS technique involving carefully timed electrical stimulation applied to peripheral nerves combined with transcranial magnetic stimulation; TMS) paradigm can be reversed into facilitation if it is first preceded by low-frequency repetitive TMS (rTMS_1 Hz_) applied to the ipsilateral dorsal premotor cortex (dPMC). Similarly, Hamada et al. (2009) have shown that the usual inhibitory LTD-like effects on M1 excitability induced by quadripulse stimulation (QPS_30 ms_; a NIBS technique involving the delivery of four precisely timed TMS pulses) can also be reversed into facilitation if preceded by low-frequency QPS_50 ms_ applied over the supplementary motor area (SMA). Similar homeostatic metaplasttic effects have been demonstrated following intra-regional priming of M1 (Siebner et al., 2004; Hamada et al., 2008).

In the current study, we investigated whether priming the left-M1 intra- or inter-regionally with cTBS can induce more consistent, and robust neuroplastic effects. The dPMC and dorsolateral prefrontal cortex (DLPFC) ipsilateral to M1 were selected as they comprise a richly interconnected network which subserves motor control (Lu et al., 1994; Picard and Strick, 2001; Fang et al., 2005; Kantak et al., 2012). Four protocols were evaluated. For intra-regional priming, active cTBS was applied to the left M1 twice. For inter-regional priming of M1, cTBS was applied to the ipsilateral (left) dPMC, and ipsilateral (left) DLPFC. A control condition was included with sham stimulation applied to ipsilateral (left) M1. It was hypothesized that intra-regional priming of M1 would suppress corticospinal excitability (Goldsworthy et al., 2012, 2015). In accordance with the BCM model of homeostatic metaplasticity, it was anticipated that inter-regional priming of the ipsilateral dPMC, and DLPFC with cTBS would facilitate M1 excitability (Hamada et al., 2009; Pötter-Nerger et al., 2009).

## Materials and Methods

### Participants

Twenty right-handed participants (*M* = 26.45, *SD* = 3.07; range = 19–33; six males) attended four sessions. Participants had no self-reported history of psychiatric, or neurological illness. Participants were screened for contraindications to TMS according to standard exclusionary criteria (Rossi et al., 2009). This study was carried out in accordance with the recommendations of the National Statement of Ethical Conduct in Human Research and approved by the Deakin University Human Research Ethics Committee (DUHREC) with written informed consent from all subjects. All subjects gave written informed consent in accordance with the Declaration of Helsinki. The protocol was approved by the DUHREC. All participants were right-handed according to the Edinburgh Handedness Inventory (*M* = 83.71, *SD* = 21.35; range = 40–100; Oldfield, 1971).

### Electromyography Recordings

Electromyography (EMG) was measured using the Trigno Wireless EMG system (Delsys, Natick, MA, USA). The electrode sensor was placed over the belly-tendon of the first dorsal interosseous (FDI) muscle of the right hand. EMG recordings (PowerLab 4/35, ADInstruments) were amplified (×1000), bandpass filtered (0.3 Hz–1 kHz), digitized (10 kHz) and epoched to the TMS pulse (−100 ms to 400 ms). Data was recorded, saved and processed offline (Labchart 7, ADInstruments).

### Single and Paired-Pulse Transcranial Magnetic Stimulation

Single-pulse transcranial magnetic stimulation (spTMS) was administered using a Magstim 200^2^ unit (Magstim, UK) via a 70 mm figure 8 coil which generates a monophasic pulse with a 100 μm rise time of 1 ms (Magstim, 2011). The coil was held tangential to the scalp at a 45-degree angle to the midline, with the lead pointing in a postero-lateral direction. The presumed motor hand region location was probed in intervals of 10 mm over the left M1 to identify the location which produced the strongest motor evoked potential (MEP) response (i.e., the hand motor “hot spot”). The test stimulus was delivered at the % maximal stimulator output (%MSO) intensity which induced an average peak-to-peak MEP of approximately 1 mV across 10 trials. Changes in corticospinal excitability were quantified as peak-to-peak MEP amplitudes before the first (priming), and after the second (test) round of cTBS. The %MSO was left unchanged for spTMS. There were five measurement points; pre-intervention (baseline), and four post-intervention time points after the second test bout of cTBS (0, 10, 20 and 30 min). For each block, 10 TMS pulses were delivered manually at a rate of 4–6 s to avoid rhythmic delivery. Paired-pulse TMS (ppTMS) was used to investigate intra-cortical inhibitory, and facilitatory mechanisms but is not reported in this paper. For ppTMS, the conditioning and test pulses were delivered with a 70 mm figure 8 coil using the Magstim Bistim^2^ (Magstim, UK) connected via the Bistim Module.

### Continuous Theta Burst Stimulation

**Figure 1:**
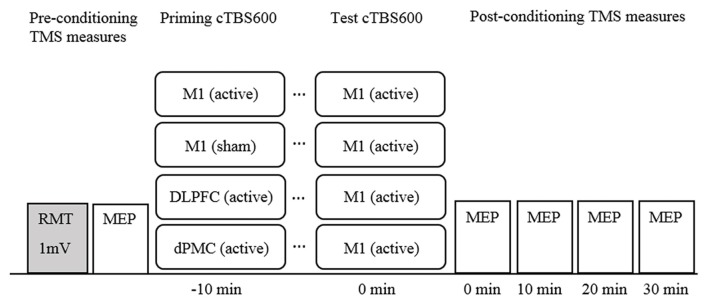
Timeline of experimental design. Note. cTBS600, continuous theta burst stimulation (600 pulses); DLPFC, dorsolateral prefrontal cortex; dPMC, dorsal premotor cortex; Ml, primary motor cortex; MEP, motor evoked potential; mV, millivolt; RMT, resting motor threshold; TMS, transcranial magnetic stimulation.

cTBS was administered using a Magstim Rapid^2^ (Magstim, UK) via a 70 mm figure 8 air cooled coil which induces a biphasic pulse of 0.5 ms duration with 80 μs rise time (Magstim, 2007a). The coil was held tangential to the scalp at a 45-degree angle to the midline for all sites, and held in place by the researcher, supported by a mechanical stand. The cTBS protocol comprised three pulses delivered at 50 Hz repeated at 5 Hz for 40 s for a total of 600 pulses at 70% resting motor threshold (RMT; cTBS600_70%RMT_; Huang et al., 2005; Goldsworthy et al., 2012). RMT for cTBS was defined as a discernible MEP of peak-to-peak amplitude greater than 50 μV in three out of five trials and was established after baseline measurements with the Magstim Rapid^2^ using a non-air cooled figure 8 70 mm coil. Sham stimulation was delivered using an identical 70 mm figure 8 air cooled placebo coil which produces a similar discharge sound, but does not stimulate the cortex (Magstim, 2007b). The experimental design is illustrated in Figure 1.

### Experimental Conditions

Participants underwent all four experimental conditions with the order of conditions determined using a randomized computer-generated sequence. Participants attended sessions at approximately the same time of day for each session. Sessions were separated by a minimum of 5 days to avoid carry over effects (Bäumer et al., 2003). The basic paradigm comprised a conditioning-test protocol with an inter-train-interval of 10 min all applied to the left hemisphere (Figure 1). This interval was selected because it has been shown to induce robust neuroplastic effects (Goldsworthy et al., 2012), but also to maintain consistency across conditions. M1 was defined functionally as the FDI “motor hotspot”. The dPMC was defined as 2.5 cm anterior to the motor hot spot on the basis of neuroimaging (Picard and Strick, 2001), and previous rTMS studies (Rizzo et al., 2004). The DLPFC was defined as 5 cm anterior to the M1 hot spot (George et al., 1995; Loo et al., 1999).

### Statistical Analysis

Only spTMS MEP data were analyzed. Outliers were defined as values exceeding absolute z-scores of 3.29. To reduce the influence of outliers, extreme values were replaced with the *M* + 3 *SD* (Field, 2009). Less than 1% of the data were transformed. Data were checked visually for normality using histograms. To better approximate normality and maintain positive values, log10 + 1 transformations were applied to raw spTMS MEP values (Tabachnick and Fidell, 2007). The primary analysis was a two-way repeated measures ANOVA with protocol (four levels: M1-M1, sham-M1, DLPFC-M1, dPMC-M1), and time (five levels: baseline, 0, 10, 20 and 30 min post-cTBS) as within-subjects factors. One-way repeated measures ANOVA (four levels: M1-M1, sham-M1, DLPFC-M1, dPMC-M1) were conducted to test for pre-conditioning differences in the %MSO 1 mV test stimulus, %MSO cTBS RMT, and baseline MEP values (transformed data). Mauchley’s test indicated that sphericity could be assumed for all analyses. Analyses were conducted offline (SPSS version 22 for Windows; SPSS Inc.). For ease of comparison, figures were generated using normalized MEP values by dividing spTMS MEP responses at each post-intervention time point (0, 10, 20 and 30 min) with pre-intervention mean baseline MEP responses. Normalized MEP values were not used for any parametric tests.

For categorical data analysis, nominal 20% post-measurement change from baseline levels were used to categorize participants as having an “expected” or “unexpected” response according to *a priori* directions of predicted change. For M1-M1 and sham-M1 conditions, participants with at least one post-intervention normalized MEP response ≤ 0.8, were classified as “expected”. For the DLPFC-M1 and dPMC-M1 conditions, participants with at least one post intervention response ≥1.2 were classified as “expected”. Cochrane’s Q analysis (exact *p*-value reported) was used to test whether the protocols differed in inducing patterns of “expected” vs. “unexpected” results. An alternative approach using the average of all post-measurement time points to categorize participants was performed and is included as Supplementary Material (refer to Data Sheet 1). Finally, individual response plots were generated for each participant across each measurement point, and protocol using normalized values.

## Results

Participants completed each session without major complaints, or adverse reactions (refer to Supplementary Figure S1). Pre-conditioning values for the %MSO 1 mV test stimulus (*F*_(3,57)_= 1.472, *p* = 0.232, *η*^2^ = 0.072), %MSO cTBS RMT (*F*_(3,57)_ = 1.728, *p* = 0.171, partial *η*^2^ = 0.083), and baseline MEP values (*F*_(3,57)_ = 1.570, *p* = 0.207, partial *η*^2^ = 0.076) did not differ across conditions (Table 1).

**Table 1:** Average (*SD*) 1 mV test stimulus and resting motor threshold (RMT) for continuous theta burst stimulation (cTBS) expressed as a percentage of maximal stimulator output (%MSO) and mean (*SD*) raw pre-conditioning motor evoked potential (MEP) values across each protocol.

	%MSO 1 mV			%MSO cTBS RMT		Pre-Conditioning MEP	
	*n*	Mean	SD	Mean	SD	Mean	SD
M1-M1	20	52.60	9.71	56.50	7.17	1.14	0.28
Sham-M1	20	55.15	9.65	58.60	9.58	1.06	0.28
DLPFC-M1	20	54.30	8.65	58.90	8.56	1.00	0.11
dPMC-M1	20	54.00	8.97	57.75	10.22	1.10	0.26

*DLPFC, dorsolateral prefrontal cortex; dPMC, dorsal premotor cortex; M1, primary motor cortex*.

**Figure 2:**
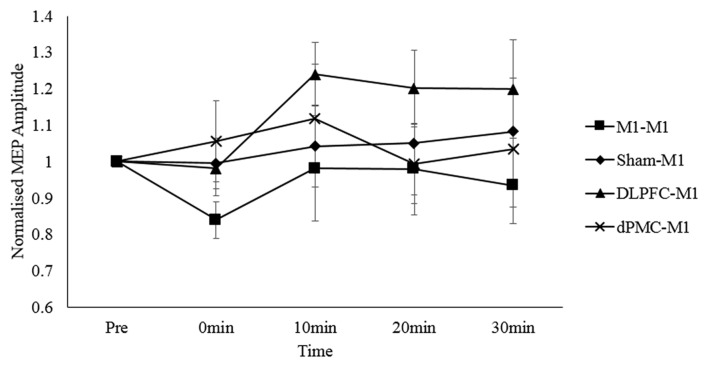
Normalized MEP amplitudes (standard errors) across each time point as a function of protocol. Note. DLPFC, dorsolateral prefrontal cortex; dPMC, dorsal premotor cortex; Ml, primary motor cortex.

For the main analysis comparing the effect of all four protocols on MEP responses, the two-way repeated measures ANOVA revealed no main effect of time, *F*_(4,76)_ = 0.683, *p* = 0.606, partial *η*^2^ = 0.035; protocol, *F*_(3,57)_ = 0.235, *p* = 0.872, partial *η*^2^ = 0.012; or time*protocol interaction, *F*_(12, 228)_ = 0.930, *p* = 0.518, partial *η*^2^ = 0.047. This indicates that M1 corticospinal excitability levels did not consistently differ between conditions (Figure 2). This was despite an increase in corticospinal excitability in the DLPFC-M1 condition from the second (10 min) post-measurement time points, and suppression in the M1-M1 condition at the first post-measurement time point.

Cochrane’s Q showed that the proportion of individuals with an “expected” vs. “unexpected” response did not differ across conditions, $\chi_{\left(3\right)}^{2}$ = 5.866, *p* = 0.136. This indicates that the protocols did not differ in inducing consistent and predicted inhibitory or facilitatory effects (Table 2).

**Table 2:** Proportion of participants classified as having an expected or unexpected response as a function of protocol.

	Expected		
Protocol	*N*	Number	Percent
M1-M1	20	14	70%
Sham-M1	20	10	50%
DLPFC-M1	20	10	50%
dPMC-M1	20	6	30%

*DLPFC, dorsolateral prefrontal cortex; dPMC, dorsal premotor cortex; M1, primary motor cortex*.

**Figure 3:**
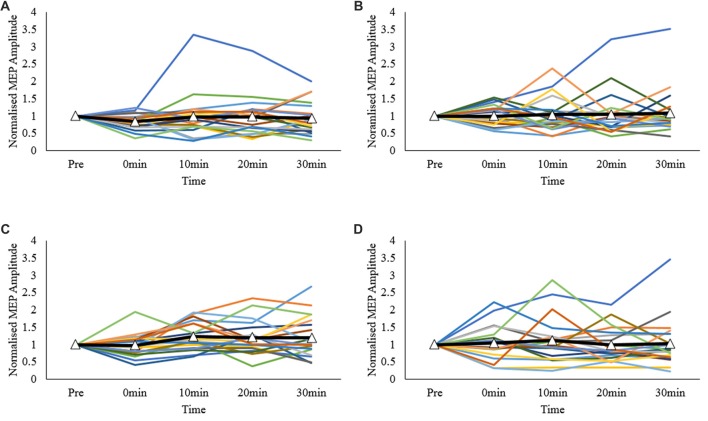
Individual MEP response plots expressed as normalized MEP amplitudes; **(A)** primary motor cortex (M1)-M1, **(B)** sham-M1, **(C)** dorsolateral prefrontal cortex-M1 (DLPFC-M1) and **(D)** dorsal premotor cortex-M1 (dPMC-M1) conditions. Bolded lines with triangle markers indicate the mean.

Normalized inter-individual values are presented in Figure 3. As observed, individual responses across each post-measurement point and protocol were highly variable.

## Discussion

The current study investigated whether intra- or inter-regional priming of the ipsilateral M1 with repeated cTBS600_70%RMT_ trains can induce homogenous, and predictable neuroplastic effects on corticospinal excitability compared to a single round of cTBS600_70%RMT_. Overall, it was found that neither intra- nor inter-regional priming of left-M1 induced robust neuromodulation of M1 corticospinal excitability. For the categorical data, the proportion of individuals with an expected inhibitory or facilitatory response did not differ across conditions. Finally, individual response plots showed that participant responses to each protocol were highly variable.

### cTBS of M1—Neurological Mechanisms

The overall null findings indicate that sham-M1 stimulation did not have significant inhibitory effects at the group level. Early studies reported that a single administration of cTBS_80%Active motor threshold (AMT)_ can induce relatively strong suppression of M1 corticospinal excitability (Huang et al., 2005). These effects are described as “LTD-like”, and have been shown to be *N-*methyl-D-aspartate receptor dependent (Huang et al., 2007; Pell et al., 2011). More recently, studies with larger samples have shown that the effects of a single administration of cTBS600_80%AMT_ can have both LTD-like and LTP-like effects (Hamada et al., 2012). Interestingly, individuals for whom TMS can more readily recruit late I-wave activity (when elicited with anterior-posteriorly induced currents) tend to show the expected suppressive effects, whilst individuals who show early I-wave recruitment tend to show facilitatory responses (Hamada et al., 2012). Future studies could examine whether differences in early- and late-I wave recruitment can be used to better predict “expected” and “unexpected” neuroplastic responses to cTBS600_70%RMT_.

### Intra-Regional Priming of M1—Neurological Mechanisms

At least four studies have examined the effects of intra-regional priming of M1 with cTBS using different inter-train-intervals, and stimulation parameters with varying results. Gamboa et al. (2011) reported that two bouts of cTBS600_80%AMT_ spaced by 20 min enhanced early suppression of M1, whilst Goldsworthy et al. (2012, 2015) showed that two rounds of cTBS600_70% RMT_ spaced by 10 min induced robust suppression of corticospinal excitability lasting up to 2 h. In contrast, Gamboa et al. (2010, 2011) found that double application of cTBS600_80% AMT_ attenuated the LTD response at short intervals (0 or 5 min), and Murakami et al. (2012) reported that sequential bouts of cTBS600_80% AMT_ at longer intervals (15 min) induced facilitation. The differences in findings are likely due to a combination of factors including different cTBS parameters (e.g., the time interval between successive bouts, the use of AMT vs. RMT), and the activation of the target muscle required to determine AMT prior to conditioning (Gentner et al., 2008).

As a whole, these findings indicate that intra-regional priming of M1 can induce either homeostatic or non-homeostatic metaplastic effects, depending on the inter-train-interval. Intra-regional priming of M1 using paired trains of cTBS600_70%RMT_ at 10 min appears to induce non-homesotatic metaplastic effects. The strong and long lasting suppression of M1 excitability for up to 2 h over and above the inhibitory effects following a single round of cTBS_70%RMT_ (Goldsworthy et al., 2012, 2015) may be due to late phase-LTD consolidation as demonstrated in animal models (Nguyen et al., 1994; Karabanov et al., 2015). In contrast, paired trains of cTBS delivered at short (0 or 5 min) or long intervals (15 min) induces facilitatory effects (Gamboa et al., 2011; Murakami et al., 2012). The reversal of the expected inhibitory effects can be explained with the BCM theory of metaplasticity (Bienenstock et al., 1982), akin to those observed in the animal literature (Abraham and Bear, 1996). Although there was some evidence of suppression of M1 corticospinal excitability in the M1-M1 condition (particularly the first post-measurement time point), the analysis failed to reach significance. Future investigations could directly compare the effect of different inter-train-intervals using a repeated measures design to better control for individual, and methodological differences.

### Inter-Regional Priming of M1—Neurological Mechanisms

Two studies examining the effects of inter-regional priming of M1 with inhibitory NIBS protocols have shown that the expected suppressive effects can be reversed in accordance with the predictions made by the BCM theory (Bienenstock et al., 1982). Pötter-Nerger et al. (2009) reported that priming the dPMC with low frequency rTMS_1 Hz_ reversed the usual inhibitory effects of paired associative stimulation (PAS_N20–5 ms_) applied over the ipsilateral (left) M1 into facilitation. Similarly, Hamada et al. (2009) reported that priming the SMA with low frequency QPS_50 ms_ reversed the usual inhibitory effects of QPS_30 ms_ applied over the left M1 into facilitation. Although visual inspection of Figure 2 shows facilitation of corticospinal excitability in the DLPFC-M1 condition, this failed to reach significance at the group level. Thus, we report that inter-regional priming of M1 with cTBS600_70%RMT_ via the ipsilateral DLPFC, or dPMC does not induce consistent neuroplastic effects.

As mentioned, the reversal of expected neuroplastic effects in previous studies are in line with the predictions made by the BCM theory of metaplasticity (Bienenstock et al., 1982; Pötter-Nerger et al., 2009; Murakami et al., 2012). Although the precise neurological mechanisms that underpin these effects in humans are unknown (Hamada et al., 2009; Pötter-Nerger et al., 2009), because the SMA and dPMC both have connections with M1 (Picard and Strick, 2001; Dum and Strick, 2005; Kantak et al., 2012) the homeostatic-like effects reported in past studies may involve cortico-cortical pathways. A limitation of the current study was MEP responses were not collected during the inter-train-interval between each round of cTBS600_70%RMT_. Furthermore, the sham-M1 protocol (i.e., the control condition) failed to induce an inhibitory effect at the group level. Nonetheless, the lack of consistent effects at the group level across all conditions makes it difficult to draw strong conclusions regarding potential neurological mechanisms induced by inter-regional priming of M1 with cTBS600_70%RMT_. However, this does not preclude the use of other protocols (e.g., iTBS), localization methods (e.g., neuroimaging), or parameters (e.g., different inter-train-intervals) in future investigations.

### Expected vs. Unexpected Responses

The categorical data analysis showed that the proportion of participants with an “expected” vs. “unexpected” response did not differ across conditions. The findings of the current study are largely consistent with the known inter-individual variability in responses following NIBS (Hamada et al., 2012). For example, both Hamada et al. (2012) and Goldsworthy et al. (2014) found that one round of cTBS600_80% AMT_ applied to the left-M1 inducted the expected inhibitory response in only 25%–30% of participants. In the current study, inter-regional priming of the DLPFC (DLPFC-M1 condition) and dPMC (dPMC-M1 condition) only induced the expected facilitatory response in 50% and 30% of participants, respectively. Thus, we report similar variability even with inter-regional priming. Goldsworthy et al. (2014) have shown that cTBS600_70% RMT_ induces inhibition in 70% of participants. In the current study, cTBS600_70% RMT_ was used for both the conditioning and test bout. We found that M1-M1, and sham-M1 stimulation induced suppression in 70% and 50% of participants, respectively. The differences in reported findings between cTBS600_80% AMT_ and cTBS600_70% RMT_ may be due to the prior activation of the target muscle when establishing AMT (Gentner et al., 2008).

### Inter- and Intra-Individual Responses

The overall null findings in the primary analysis is most likely driven by the high degree of intra- and inter-individual variability between protocols and across each measurement time point. Human responses to NIBS are known to be highly variable (Ridding and Ziemann, 2010; Hamada et al., 2012; Suppa et al., 2016). In one of the largest single studies to date (*n* = 57), Hamada et al. (2012) showed that a single application of cTBS, or intermittent TBS (iTBS) to the left-M1 resulted in highly variable neuroplastic responses. So much so, there were no overall differences at the group level. In the current study, it was also found that participant responses to intra- and inter-regional priming of left M1 using cTBS600_70% RMT_ were highly variable. The factors which contribute to such variability remain to be elucidated but may include the prior activation history of a target region (Gentner et al., 2008), genetic factors (Cheeran et al., 2008), intrinsic fluctuations in corticospinal excitability (Kiers et al., 1993), individual differences in inter-neural networks (Hamada et al., 2012), and inherent variability in human responses to NIBS (Ridding and Ziemann, 2010).

## Conclusion

The current investigation found that neither intra- nor inter-regional priming of the left M1 with cTBS600_70%RMT_ induced consistent, and robust effects on corticospinal excitability. A high degree of inter-individual variability was observed regardless of whether the prime was applied inter- or intra-regionally. The neural mechanisms underpinning these findings are not clear. Potential mechanisms may include LTD-like mechanisms, or homeostatic and non-homeostatic metaplasticity. Further work is required to better understand the factors contributing to this variability. This in turn can be used to better inform research, and clinical parameters involving priming protocols.

## Author Contributions

All authors named in this publication contributed equally to the design, implementation, and collection of data. Each author also contributed to the drafting, revision and preparation of the manuscript. All authors provided approval for final manuscript to be submitted for publication.

## Conflict of Interest Statement

The authors declare that the research was conducted in the absence of any commercial or financial relationships that could be construed as a potential conflict of interest.
