# Supplementary material for: Intra- and Inter-Regional Priming of Ipsilateral Human Primary Motor Cortex With Continuous Theta Burst Stimulation Does Not Induce Consistent Neuroplastic Effects
Source: Front Hum Neurosci. 2018 Mar 29;12:123. doi: 10.3389/fnhum.2018.00123 (PMC5884878; doi:10.3389/fnhum.2018.00123)
Supplement: Supplementary file 1 [file Table_1.docx]

Supplementary Material

**Intra- and Inter-Regional Priming of Human Primary Motor Cortex with Continuous Theta Burst Stimulation Induces Variable Homeostatic and Non-Homeostatic Metaplastic-Like Effects**

**Mr Michael Do*, Dr Melissa Kirkovski, Ms Charlotte B. Davies, Ms Soukayna Bekkali, Dr Linda K. Byrne, Professor Peter G. Enticott**

*** Correspondence:** Michael Do: m.do@deakin.edu.au

**Supplementary Table 1**

Frequency (n) and proportion (%) of participants who reported experiencing the following adverse events as a function of protocol.

|  | M1-M1 | |  | Sham-M1 | |  | DLPFC-M1 | |  | dPMC-M1 | |
| --- | --- | --- | --- | --- | --- | --- | --- | --- | --- | --- | --- |
| Adverse event | Frequency | (%) |  | Frequency | (%) |  | Frequency | (%) |  | Frequency | (%) |
| Ear Pain | 0 | (0) |  | 0 | (0) |  | 0 | (0) |  | 1 | (5) |
| Face pain | 2 | (10) |  | 0 | (0) |  | 3 | (15) |  | 1 | (5) |
| Fatigue | 2 | (10) |  | 2 | (10) |  | 2 | (10) |  | 2 | (10) |
| Headache | 3 | (15) |  | 1 | (5) |  | 2 | (10) |  | 6 | (30) |
| Migraine | 0 | (0) |  | 1 | (5) |  | 0 | (0) |  | 0 | (0) |
| Neck pain | 2 | (10) |  | 2 | 10) |  | 3 | (15) |  | 2 | (10) |
| Nervousness | 0 | (0) |  | 1 | (5) |  | 0 | (0) |  | 0 | (0) |
| Numbness | 0 | (0) |  | 0 | (0) |  | 1 | (5) |  | 1 | (5) |
| Scalp pain | 3 | (15) |  | 2 | (10) |  | 6 | (30) |  | 5 | (25) |
| Shoulder Pain | 0 | (0) |  | 1 | (5) |  | 0 | (0) |  | 0 | (0) |
| Tingly | 3 | (15) |  | 1 | (5) |  | 2 | (10) |  | 3 | (15) |
| Toothache | 0 | (0) |  | 1 | (5) |  | 0 | (0) |  | 0 | (0) |
| Weepiness/crying | 0 | (0) |  | 0 | (0) |  | 1 | (5) |  | 1 | (5) |

*N = 20 for all protocols. One participant experienced a transient, migraine 15min after the end of one session (DLPFC-M1 condition) that resolved spontaneously after 30min. DLPFC – dorsolateral prefrontal cortex; dPMC – dorsal premotor cortex; M1 – primary motor cortex.*

**
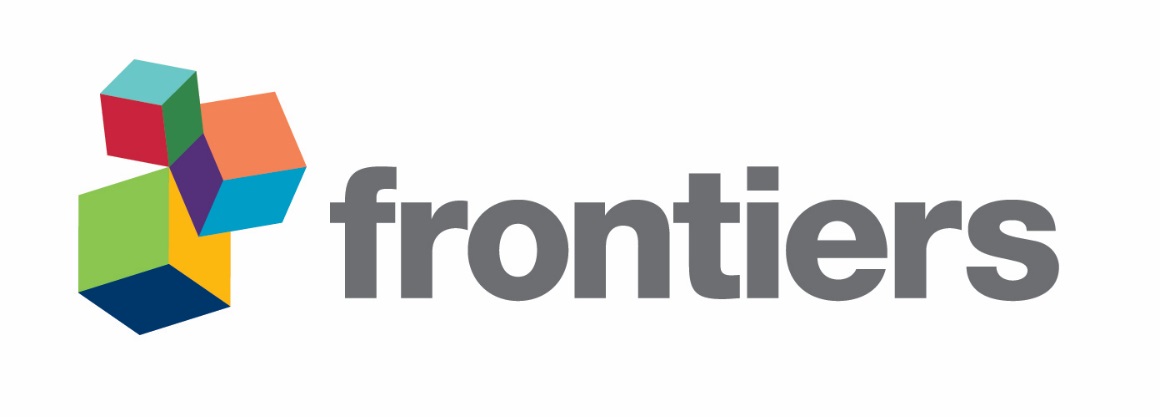
**
