# Supplementary material for: Intra- and Inter-Regional Priming of Ipsilateral Human Primary Motor Cortex With Continuous Theta Burst Stimulation Does Not Induce Consistent Neuroplastic Effects
Source: Front Hum Neurosci. 2018 Mar 29;12:123. doi: 10.3389/fnhum.2018.00123 (PMC5884878; doi:10.3389/fnhum.2018.00123)
Supplement: Supplementary file 2 [file Data_Sheet_1.docx]

**Statistical analysis**

For categorical data analysis, the average across all post-measurement time points were collapsed and used to classify participants as having an “expected” or “unexpected” response according to *a priori* directions of predicted change. For M1-M1 and sham-M1 conditions, participants with average normalized MEP post-intervention responses ≤.8, were classified as “expected”. For the DLPFC-M1 and dPMC-M1 conditions, participants with average normalized post-intervention MEP responses ≥1.2 were classified as “expected.” Cochrane’s Q analysis (exact *p*-value reported) was used to test whether the protocols differed in inducing patterns of “expected” versus “unexpected” results. Finally, individual response plots were generated for each participant across each measurement point and protocol using normalized values.

**RESULTS**

Cochrane’s Q showed that the proportion of individuals with an “expected” versus “unexpected” response did not differ across conditions, *χ*^2^ (3) = 2.800, *p* = .500. This indicates the protocols did not differ in inducing consistent and predicted inhibitory or facilitatory effects (Table 2).

TABLE 2. Proportion of participants classified as having an expected or unexpected response as a function of protocol.

|  |  | Expected | |
| --- | --- | --- | --- |
| Protocol | *N* | Number | Percent |
| M1-M1 | 20 | 9 | 45% |
| Sham-M1 | 20 | 4 | 20% |
| DLPFC-M1 | 20 | 8 | 40% |
| dPMC-M1 | 20 | 7 | 35% |

DLFPFC – dorsolateral prefrontal cortex; dPMC – dorsal premotor cortex; M1 – primary motor cortex.

**DISCUSSION**

**Expected versus Unexpected responses**

The categorical data analysis showed that the proportion of participants with an “expected” versus “unexpected” response did not differ across conditions. The findings of the current study are largely consistent with the known inter-individual variability in responses following NIBS (Hamada et al., 2012). For example, both Hamada et al. (Hamada et al., 2012) and Goldsworthy et al. (Goldsworthy et al., 2014a) found that one round of cTBS600_80% AMT_ applied to the left-M1 inducted the expected inhibitory response in only 25-30% of participants. In the current study, inter-regional priming of the DLPFC (DLPFC-M1) and dPMC (dPMC-M1) only induced the expected facilitatory response in 40% and 35% of participants, respectively. Thus, we report similar variability even with inter-regional priming. Goldsworthy et al. (2014a) have shown that cTBS600_70% RMT_ induces inhibition in 70% of participants. In the current study, cTBS600_70% RMT_ was used for both the conditioning and test bout. We found that M1-M1 and sham-M1 stimulation induced suppression in 45% and 20% of participants, respectively. The differences in reported findings between cTBS600_80% AMT_ and cTBS600_70% RMT_ may be due to the prior activation of the target muscle when establishing AMT (Gentner et al., 2008)_._
